# Guidelines: The dos, don’ts and don’t knows of remediation in medical education

**DOI:** 10.1007/s40037-019-00544-5

**Published:** 2019-11-06

**Authors:** Calvin L. Chou, Adina Kalet, Manuel Joao Costa, Jennifer Cleland, Kalman Winston

**Affiliations:** 1 Department of Medicine, University of California and Veterans Affairs Healthcare System, San Francisco, CA USA; 2 Department of Medicine, New York University School of Medicine, New York, NY USA; 3 Life and Health Sciences Research Institute, School of Medicine, University of Minho, Minho, Portugal; 4 Centre for Healthcare Education Research and Innovation (CHERI), University of Aberdeen, Aberdeen, UK; 5 Department of Public Health and Primary Care, Cambridge University, Cambridge, UK

**Keywords:** Remediation, Feedback, Struggling learner, At-risk students

## Abstract

**Introduction:**

Two developing forces have achieved prominence in medical education: the advent of competency-based assessments and a growing commitment to expand access to medicine for a broader range of learners with a wider array of preparation. Remediation is intended to support all learners to achieve sufficient competence. Therefore, it is timely to provide practical guidelines for remediation in medical education that clarify best practices, practices to avoid, and areas requiring further research, in order to guide work with both individual struggling learners and development of training program policies.

**Methods:**

Collectively, we generated an initial list of Do’s, Don’ts, and Don’t Knows for remediation in medical education, which was then iteratively refined through discussions and additional evidence-gathering. The final guidelines were then graded for the strength of the evidence by consensus.

**Results:**

We present 26 guidelines: two groupings of Do’s (systems-level interventions and recommendations for individual learners), along with short lists of Don’ts and Don’t Knows, and our interpretation of the strength of current evidence for each guideline.

**Conclusions:**

Remediation is a high-stakes, highly complex process involving learners, faculty, systems, and societal factors. Our synthesis resulted in a list of guidelines that summarize the current state of educational theory and empirical evidence that can improve remediation processes at individual and institutional levels. Important unanswered questions remain; ongoing research can further improve remediation practices to ensure the appropriate support for learners, institutions, and society.

## Definitions of dos, don’ts, and don’t knows

### Do’s

Educational activity for which there is evidence of effectiveness

### Don’ts

Educational activity for which there is evidence of no effectiveness or of harms (negative effects)

### Don’t knows

Educational activity for which there is no evidence of effectiveness

## Introduction

Remediation in medical education is ‘*the act of facilitating a correction for trainees who started out on the journey toward becoming a physician but have moved off course*’ [1]. In the past, when encountering struggling learners, medical educators had little guidance on how to support or intervene effectively to ensure competence or make promotion judgments. In recent years, in response to frustration with the piecemeal approach to remediation and its potentially unacceptable consequence of graduating physicians not ready to practise safely, there has been a dramatic growth in the literature on remediation in medical education.

Reports of the cumulative prevalence of trainees in need of remediation have ranged from 2.0% in surgical residencies [2] to 3.3% in medical school [3]. The reported success of remediation has ranged from 77% [2] to 100% [3–5]. However, there is no standard definition of ‘success’, and most programs report only short-term outcomes. For example, one report showed that while 91% of students passed the first semester after remediation, only 61% had completed the entire program 2 years later [6]. Additionally, the criteria programs use to identify learners needing remediation vary widely, even within the same institution.

Not surprisingly, as demands on trainees shift over the course of medical training, the types of remediation challenges change. Early medical students tend to struggle with knowledge and skills gaps, ways of thinking, self-regulation, and approaches to learning [7]. In addition to insufficient knowledge, students in clerkships can struggle with patient presentation skills, foundational communication skills (e.g. introducing oneself), physical examination skills, and the application and synthesis of knowledge to create individualized patient plans [8, 9]. For residents and foundation years (the 2 years immediately after medical school in the UK), knowledge can continue to be a major area of struggle [5, 10, 11]. In addition, learners at these stages of medical training can manifest difficulty with clinical judgment [2, 5, 10, 11], communication [5], professionalism [2, 4, 5, 11, 12], time management, and organization skills [5, 10, 13].

Medical students and physicians are not accustomed to struggling. Selection into medical school requires high academic ability, and medical students are used to achieving. Consequently, when faced with academic failure, many may experience disproportionate emotional reactions that can exacerbate the problem and limit their ability to adapt quickly and focus on remedial work. In some settings, there may be a significant economic burden of failure (e.g. retaking exams or courses) which is mostly borne by the student [14] or the program [15]. We also know that some learners minimize, externalize, and blame faculty and the institution for their struggles [16, 17], making it even more challenging for supervisors and institutions to provide effective remediation.

Medical education and training programs must navigate competing interests surrounding identification and remediation of struggling learners. Generally, educators may feel tremendous responsibility for, and often identify with, learners, particularly when such a great deal of time, resources, ego, and energy has been invested into medical training. Further, the presence of a struggling learner requires increased monitoring, counselling, and other costly remediation strategies, which may tax program and faculty resources. It may also damage the integrity of the program or negatively influence the experience of peers [18–20]. In addition, the well-documented ‘failure to fail’ in medical education is troubling because it challenges the social contract medicine has with society by erring toward keeping marginally competent practitioners in the profession [21–23]. It is common to give struggling learners repeated marginal passes that avoid addressing the underlying problems [16, 24, 25]. Programs sometimes inadequately reassess learners in remediation, failing to ensure remediation was successful [26]. Ultimately, however, the medical profession has a responsibility to ensure that it will graduate learners that fulfil its social responsibility for high quality, safe, professional care [27].

In sum, faculty members must possess the confidence, knowledge of systems and standards, motivation, and self-efficacy to recommend a struggling learner for remediation, in part because this decision must be defended to all stakeholders, including the learners themselves, peers, program leadership, and society [22, 28]. Medical curricula must create learning environments that support all students to thrive [29, 30]. This is especially important as the profession works to increase access to medical careers for traditionally underrepresented populations. Having suffered structural educational discrimination, these groups may need extra support when entering a medical culture slow to change norms and values around learning [31]. Moreover, institutions, concerned about legal consequences from trainees and future patients [21], may intentionally avoid having official policies on remediation and probation [32]. These phenomena, which together contribute to overall institutional culture, create barriers to effective identification of, and intervention with, struggling learners, most of whom will soon be (or are already) practising physicians.

We thus present these guidelines with the aim of aiding the development of remediation practice. The guidelines are divided into two highly interrelated groupings: *system *and* individual level guidelines* [18, 20, 33].

## Methods

These guidelines are based on consensus of expert opinion across medical educators based in four countries who have published scholarship in this area, supplemented with a targeted review of the literature on remediation in medical education. We address the continuum of medical education from beginning medical school to certification as an independent clinician at the end of specialty training. Sensitive to the fact that training differs somewhat across countries, we aim to provide guidelines that are relevant across contexts. We also believe these guidelines may be applicable across healthcare professions, even though our main experience is with training physicians.

We utilized an iterative process similar to that outlined in previously published papers for this *Guidelines* series [34]. Following agreement upon the definition provided above, AK shared an initial list of Do’s, Don’ts, and Don’t Knows. Each author, drawing upon their own scholarship, personal experience and understanding of the relevant literature, added to this list. The combined list was then consolidated and categorized, initially by CC and MC, with further input from the other authors. Next, through a series of discussions via email and Skype®, the lists were reviewed, discussed and refined until reaching consensus on the Do’s, Don’ts, and Don’t Knows. We sought to harmonize terminology so that it would be understood across countries. Many ‘Don’ts’ on the original lists were acknowledged to be simply negations of some ‘Do’s’—these were removed to avoid unnecessary repetition. When evidence was conflicting or there was no clear consensus among us for Do’s or Don’ts, the item was categorized in the Don’t Know section—we consider these to be important areas for further research.

CC then conducted a targeted literature review of the literature on remediation in medical education, producing a final organized list of Do’s, Don’ts, and Don’t Knows in a first draft of this paper. All authors then contributed further comments, evidence, and edits. Subsequently, all offered their independent opinions on the strength of evidence for each guideline and reached consensus on the rating for each guideline (Tab. 1). We further refined the list after suggestions from journal editors.

**Table 1:** Criteria for strength of recommendation

Strong	A large and consistent body of evidence
Moderate	Solid empirical evidence from one or more papers plus consensus of the authors
Tentative	Limited empirical evidence, but clear consensus of the authors

## Results (Tab. 2)

**Table 2:** Summary of guidelines for remediation in medical education

Guideline	*Systems level, Do’s*	*Recommendation*
1	Do advertise to the entire medical education community that learners commonly need remediation, which is resourced and available to all learners	Tentative
2	Do develop a robust feedback culture that impels learner improvement	Moderate
3	Do align selection and assessment systems with desired outcomes and graduate qualities	Strong
4	Do construct strategies aimed at averting the need for remediation	Strong
5	Do deliver remediation as highly individualized processes while recognizing common patterns across struggling learners	Moderate
6	Do ‘feed forward’ remediation information, with an abundance of caution	Moderate
7	Do provide faculty development and tangible support for frontline educators in early identification of, effective interventions for, and appropriate referral of struggling learners	Tentative
8	Do separate the individuals conducting the remediation process from those who determine the outcome of remediation	Tentative
9	Do ensure due process, balancing empathy for individual students’ struggles with the medical profession’s responsibility to society	Moderate
10	Do create compassionate alternative pathways for those who do not choose to or cannot complete medical training	Tentative
	*Remediation process, Do’s*	
11	Do aim to detect a need for remediation early	Moderate
12	Do collect relevant data from multiple sources across case content	Strong
13	Do explore multiple causes of learner struggle beyond educational or workplace issues	Strong
14	Do intervene proactively with struggling learners—do not rely on their initiative	Strong
15	Do have trainees in remediation undergo intensive, longitudinal tutoring with emphasis on study skills, collaboratively designed plans, frequent high-quality feedback, and individualized assessment	Strong
16	Do assess for and improve skills in learning self-regulation	Strong
17	Do remediate knowledge and skills in small groups with expert facilitators	Moderate
18	Do follow up with learners, even after the presumed end of the remediation period	Moderate
	*Don’ts*	
19	Don’t rely solely on traditional academic markers of performance	Moderate
20	Don’t merely give more time, repeat the learner experience, give general or vague advice, or just ‘teach to the test’ without additional support	Strong
	*Don’t knows*	
21	What are the long-term outcomes of remediation?	
22	What is the optimal blend and duration of remediation?	
23	How does remediation fit with CBME and its approach of learner-centredness and de-emphasis of time?	
24	What is the optimal balance between the benefits of educational handovers and the need to protect learners from negative bias that may arise from such handovers?	
25	What specific measures predict the need for remediation?	
26	Apart from establishing a longitudinal remediation program (Guideline 15), what are the most effective remediation practices?	

### Part I. System level/Contextual issues

We agree with Steinert’s statement: *‘Many potentially difficult situations can be prevented by setting expectations, giving feedback, and providing thoughtful, ongoing evaluation’ *[18]. We note that it is common for learners to struggle partly because the educational system has failed them. We assert that, in many cases, the underlying cause of learner struggles is situated in a series of misalignments between them and the learning context, akin to how patient care errors may arise from the interface between human and systems failures [35]. For example, when a student fails a knowledge exam or a clerkship, it is rarely helpful to treat it as an isolated event addressed by a quick fix, such as teaching to a specific exam [7, 17, 36]. Remediation is most successful when based on an analysis to detect patterns of maladaptive development or alignment. Though remediation work typically focuses on the individual learner, it should also ideally feed back to the program and lead to adjustments that ultimately benefit a larger group of learners.

#### Guideline 1.

*Do advertise to the entire medical education community that learners commonly need remediation, which is resourced and available to all learners ***(tentative)**

Whether or not an institution openly acknowledges the predictable need for remediation and works to destigmatize and adequately resource remediation in medical education is a reflection of the culture of that institution [37]. Medical education programs have traditionally used deficit-based approaches to education, which can encourage learners to focus on surface performance rather than deeper understanding to avoid negative labels [38]. More recently, programs have increased adoption of competency-based approaches, which de-emphasize time spent in training and emphasize the developmental, possibly time-variable, nature of the acquisition of capacities [39]. In this model, many, if not most, students might need support at one time or another [27]. Indeed, we advocate explicitly reframing, and thereby destigmatizing, remediation as a special zone of learning, self-improvement, personal development, resilience building, and an opportunity to practise with feedback, all to develop the adaptive capacity needed by all medical professionals in the current era [27, 40, 41]. This goal requires that program leadership and faculty embody this approach in substantial ways. Adopting a culture in which ‘educational alliances’ are formed with learners, where there is unconditional positive regard for the person rather than taking a deficit-based approach, can support a growth mindset [42–44].

An institutionally-based programmatic approach normalizes remediation. For example, one school increased the percentage of students attending voluntary remediation sessions from 40% to nearly 70% when it instituted a transparent academic policy that required satisfactory completion of tasks and facilitated early identification and support of struggling students [45]. Accordingly, we strongly encourage programs to explicitly advertise an expectation that learners may require remediation services and to direct learners on how to access these services.

#### Guideline 2.

*Do develop a robust feedback culture that impels learner improvement*** (moderate)**

The importance of a robust feedback culture is one of the best-documented aspects of effective education [34, 46–48]. All learners benefit from close observation, effective feedback, and ongoing formative assessment [34]. Immersing learners fully and actively in ongoing feedback processes increases their motivation and engagement in the lifelong learning that characterizes ideal medical practice [31, 49]. Identifying how best to support clinical teachers in delivering feedback to students has been a focus of research activity for many years [50, 51]. Unfortunately, students consistently report dissatisfaction with the feedback they receive [52–54].

Multisource feedback enhances the impact of recommendations for improvement. In a randomized controlled study of multisource feedback on communication skills and professionalism, paediatrics residents performed self-assessments, received reports of parent and nurse evaluations of their skills, and underwent tailored coaching. Nurse ratings of residents’ communication skills, timeliness, and demonstration of responsibility and accountability increased for residents receiving multisource feedback and decreased for the control group [55].

Establishing a trustworthy, dialogic, learner-focused and transparent programmatic feedback culture [56, 57], where all members of the medical education program are trained to give, receive, expect and respect feedback from all other members of the program, is especially important in helping struggling learners attain and maintain performance improvements.

#### Guideline 3.

*Do align selection and assessment systems with desired outcomes and graduate qualities*** (strong)**

As mentioned earlier, remediation involves more than intervening at an individual level. Globally, there is increasing interest in incorporating groups who have not traditionally participated in medical education and therefore may need help transitioning to professional education. Increasing the diversity of students has highlighted the need to align selection, assessment, and support systems, to ensure a culture of support in medical education rather than one of ‘sink or swim.’

Selection is the first, and perhaps most important, assessment in medical education [58]. Developing a fair and accurate selection process capable of identifying applicants based on the necessary academic and interpersonal, ‘noncognitive’ criteria is challenging [59, 60]. This is likely best achieved by defining the competencies of a ‘good doctor’ and using them as the basis of an outcome-based selection procedure [61–63].

All subsequent assessments, curricula and teaching must incorporate high degrees of fairness, reliability, and validity, and should ideally align with the desired knowledge, attitudes, and behaviours expected from graduates, and ultimately practising clinicians [64, 65].

#### Guideline 4.

*Do construct strategies aimed at averting the need for remediation*** (strong)**

In addition to our arguments in the above section for robust explicit educational systems, there is strong evidence for two specific strategies that can potentially prevent the need for remediation: retrieval rehearsal and support groups.

Frequent quizzing, or retrieval rehearsal, improves performance on knowledge exams [66, 67]. In addition to strong evidence in support of retrieval rehearsal on the retention of knowledge from a wide range of settings [68–70], one study showed that residents who were tested with multiple choice questions before and after didactic lectures improved their performance on an otolaryngology in-training examination. [71].

Many stressors, particularly those fostered by the implicit curriculum, can influence the need for future remediation. Students report feeling that they are constantly being evaluated, particularly in the setting of clerkships where the criteria and expectations for grades are subjective and appear arbitrary and whimsical [38, 72, 73]. A strategy shown to have potential to alleviate some of that stress is the facilitated support group with sessions aimed at increasing self-awareness, self-care, and/or mindfulness training [74–76].

#### Guideline 5.

*Do deliver remediation as highly individualized processes while recognizing common patterns across struggling learners*** (moderate)**

Because learner difficulties have a multitude of causes and manifestations, a single course/program director or remediator, while essential to guide and coordinate remediation, cannot adequately synthesize all the skills necessary to conduct impactful remediation. Therefore, the responsibility for effective remediation lies with teams that include complementary expertise and institutional roles. Remediation teams, assembled *ad hoc* for a particular learner, may comprise faculty with deep expertise in particular areas (e.g., communication skills, clinical reasoning), learning specialists, standardized patient trainers, mental health professionals, public speaking coaches, among many others [77, 78]. The team-based approach can allow for the highly customized, multi-pronged remediation that struggling learners need to succeed.

However, there are some common patterns to the struggles of medical learners (see Guideline 13) that can be addressed efficiently in groups. Both individualized and group remediation experience should inform curricular and systems improvements for all students.

#### Guideline 6.

*Do ‘feed-forward’ remediation information, with an abundance of caution*** (moderate)**

Learner handover, sometimes termed ‘feeding forward’, is a controversial area of remediation practice where subsequent course directors or clinical supervisors receive information about struggling learners. The main detractors of this practice, which include some learners, prioritize their concerns that learners may suffer from stigmatization and bias, explicit or implicit, that result in unfair treatment [79]. On the other hand, others argue that lack of continuity of information hinders the early identification of struggling students and the remediators’ ability to intervene effectively [25, 29, 80–83]. The approach to learner handover appears to be highly variable. For example, approximately half of US schools claim that they do (or don’t) engage in the practice, and many schools lack a formal policy on this issue [84]. Though privacy concerns about learner handovers may arise, in some settings, university officials with legitimate educational interests may legally engage in such activities without explicit student consent [85–87].

There is evidence for both potential harms and benefits of educational handovers about struggling learners, making the judgment about doing so complex. A recent scoping review evaluating learner handovers in a wide variety of mostly nonmedical settings, suggests that prior information about performance biases subsequent ratings, with some evidence that negative prior information may exert a larger effect than positive prior information [88]. However, using specific performance standards to share information may mitigate bias in subsequent judgments [89]. As far as we have been able to ascertain, there has been no legal case raised in the US against sharing information about a struggling learner for educational purposes. Indeed, this is common practice in other countries, including the UK. Moreover, when program leaders of a pre-clerkship curriculum feed-forward information about students to the subsequent module leader, it helps identify struggling students, reduces ‘failure to fail’ by distributing responsibility for failing a student, reduces concerns about some students, and increases the detection of professionalism problems [24]. Finally, simply sharing information about students at risk (e.g. from low-income backgrounds) resulted in more resources for struggling learners and improved grades [90].

We believe that the learner handover is a practice which is in most cases justified by our profession’s social contract. We therefore recommend that programs formally institutionalize this practice with strong caveats: that it occurs with the student’s knowledge and sensitivity to the student’s privacy, consists of low-inference information based in specific performance standards that are likely to lead to an effective remediation strategy, and is shared only with other members of the faculty who can support the remediation goals (see Guideline 24).

#### Guideline 7.

*Do provide faculty development and tangible support for frontline educators in early identification of, effective interventions for, and appropriate referral of struggling learners ***(tentative)**

Faculty remediators generally have low confidence in their ability to conduct remediation [91], particularly in professionalism [92]. Many faculty are unsure about identifying what is permissible and what does not meet the standard [22], while more experienced faculty likely remediate more effectively [93, 94].

Remediation requires much more time, expertise and resources than most faculty allocate or expect [95, 96]. One study placed the number of faculty hours needed to address efficiency and organizational skills at 25–75 hours per struggling resident [13]. We believe that remediation cannot be done solely on a voluntary basis and should be done by highly experienced people who are remunerated adequately.

Faculty development for remediation requires both individually and institutionally focused capacity-building processes. A set of specific competencies, attributes of teachers, theories of learning, and teaching strategies specific to remediation work have been proposed [97]. In particular, effective faculty remediators must be able to judge the performance of medical learners across a full range of competencies, develop facilitation skills, and cultivate emotional intelligence, courage, and attitudes consistent with effective remediation work. We recommend programs to build faculty development processes that nurture a community of practice of select, highly motivated educators that develop specialized domains of expertise. This community should integrate with other important communities of practice (e.g., education and workplace), where all medical educators develop a focused set of skills (e.g., identifying and referring the struggling learner to remediation, cultivating the feedback culture and expertise).

#### Guideline 8.

*Do separate the individuals conducting the remediation process from those who determine the outcome of remediation***(tentative)**

Medical education promotion or dismissal judgments are complex and highly consequential. Ideally, experienced faculty make these decisions dispassionately, after careful contextual review of the learner’s performance against stated expectations. Invariably, significant uncertainty exists in the available data.

Remediators must personally engage with the struggling learner in order to establish trust, confidentiality, and boundary limits [37, 41]. Therefore, to avoid inherent conflicts of interest and make defensible judgments, those conducting the intimate remediation with the trainee *must not* be the same people who make the final adjudication decisions [37, 98]. This can be a challenge in small programs with few faculty members with the necessary experience. In such cases, program directors can establish *ad hoc* committees of faculty from other similar programs to remediate or make final promotion decisions. Accreditation requirements in some parts of the world may offer policy and procedural guidance.

#### Guideline 9.

*Do ensure due process, balancing empathy for individual students’ struggles with the medical profession’s responsibility to society ***(moderate)**

Learners, particularly some who most need help, commonly do not like to expose themselves and may fear they have been labelled, perhaps unfairly, as ‘struggling’. It can be helpful for the embarrassed learner to know that any individual’s privacy will be protected [6, 83, 99]. The process must be fair and confidential and include informed consent, in that the learner should know as much about the processes and potential outcomes, including dismissal, as possible. Institutional policies must support careful documentation and communication among team members delineating the decision and the reasons for remediation, a written individualized remediation plan (with goals, instructional strategies, and assessments) as well as the sequence of events, and who is responsible for each remediation area, reporting structure, time frame, and decision making [22]. Supporting data are key, including the intervention plan and learning contract, observed outcomes, and ongoing summaries of discussions with learners and colleagues [37]. While there is a responsibility to ensure fair process for the individual trainee, it is equally important that medical education fulfils its contract with society to produce competent physicians [37, 98].

#### Guideline 10.

*Do create compassionate alternative pathways for those who do not choose to or cannot complete medical training ***(tentative)**

While most would agree that not every learner who is admitted can, or should, graduate from the health profession program they start, there is great regional variation in how this manifests and impacts remediation policy and practice. For example, the remediation goals of a medical school in a system where not everyone who starts is expected to graduate (e.g., Switzerland) differ from schools in the UK, where the number of medical school enrolments is controlled by the government on the basis of national workforce planning forecasts. However, whilst taking context into account, an important role of remediation is to enable a realistic assessment of the likelihood of long-term success, encourage honest learner self-reflection, and offer other viable options. Some experienced educators believe that some struggling students, particularly those who felt pressure from family to enter the profession, may ‘self-sabotage’ as a face-saving way to change career path. Especially important in the US, but relevant in many regions, restructuring or forgiving financial debt may enable students who cannot or do not choose to continue to train as physicians to leave medical training without crushing financial and/or personal consequences. Counselling on viable options for alternative career paths must be developed and made available to all medical trainees. Studies of alternate pathways taken in countries where many who enter medical school do not finish would inform the development of such policies elsewhere [100, 101].

## Part II. The remediation process

While the systems-level guidelines above are critically important, ultimately the remediation process is highly individualized to the needs of the particular struggling learner. For remediation to be effective, the learner must be identified; the areas of struggle clarified; underlying causes or explanations explored and understood [18]; a flexible remediation intervention crafted [3, 90, 102, 103] and implemented; and progress assessed. Responsibility for remediation starts with course or program directors. The work of the clarification and intervention is mostly conducted within the remediator-struggling learner dyad. Finally, the responsibility returns to course or program directors for assessment of outcome. Ultimately a disposition judgment needs to be made (see Guideline 8).

The centrepiece of excellent remediation responses is establishment of an appropriate, achievable intervention or learning plan that directly addresses the deficiencies via skilled feedback [34], often based on direct observation in clinical settings [104]. However, remediation plans for individual struggling learners never exist in isolation. As previously mentioned, remediation exerts emotional impacts on and resource costs to other individuals in the program. For example, remediation interventions for one learner may be perceived by peers as unfair special treatment, especially if it requires those peers to assume extra duties to cover the time needed for the struggling learner’s remediation activities. We recommend that program leadership provides clear, supportive, empathic anticipatory guidance to peers of struggling learners, while maintaining respect for the remediating learner’s privacy. In our experience, this approach helps peers rise to challenges and gain a sense of positive, inspired, and supportive camaraderie. Finally, since resources to undertake remediation efforts are almost always limited, institutions and course/program directors must remain strategic in deployment, intervention, and evaluation (Guidelines 1 and 9).

### Guideline 11.

*Do aim to detect a need for remediation early***(moderate)**

Early identification of learners who struggle in many competency areas can maximize the success of remediation interventions. For knowledge deficits, early struggle on any assessment in the pre-clerkship phase of medical school predicts later underperformance [22, 105–107]. Analysis of struggling students at one UK medical school suggested that a combination of predictors, including performance on examinations, unprofessional behaviour, health problems, social problems, and missed required vaccinations, may augur the need for remediation in the pre-clerkship phase [108]. At another UK medical school, when early identification of struggling pre-clerkship students occurred at 4, 7, and 12 months after starting, more students participated in supportive services, compared with historical controls, and those engaging in one-on-one remediation services were more likely to successfully complete pre-clerkship studies [45].

There is some literature that defines parameters that influence early identification of struggling students in clerkships. Pre-clerkship knowledge and early clinical performance predict workplace-based clinical performance in medical schools in the UK [97], the Netherlands [105], and United Arab Emirates [109]. Low clerkship ratings and lack of student progress on communication skills or professionalism concerns predict failure on the patient-provider interaction portion of a high-stakes clinical skills examination given at the end of foundational clerkships [110]. Students referred for remediation after their internal medicine clerkship were more likely to receive poor ratings in internship and fail USMLE Step 3 [111]. Guerrasio reported that three medical students with previously-identified interpersonal skills deficits did not match into any residency program and therefore could not continue their medical training [3].

Professionalism lapses predict future struggles in medical school and probably much more. Papadakis [112] showed that while low MCAT scores (pre-medical school) and low grades during the first 2 years of medical school carry a 7% risk of subsequent disciplinary action as practising physicians, identification of unprofessional behaviour in that same period increases this risk to 26%. In a pre-clerkship curriculum, three or more unexcused absences from attendance-required sessions and negative peer assessment correlate with unprofessional conduct during clinical years [113]. While personality measures seem to have little power to predict academic struggles [114, 115], there may be an association between behaviour and personality. Physicians who demonstrated unprofessional behaviour during medical school scored lower on four out of six scales of the California Psychological Inventory [116]. Finally, poor professionalism was the only statistically significant predictor for placement of clinically-based learners or practitioners on official probationary status at one US institution [3].

This literature supports early identification of students who struggle with learning medical knowledge, patient care, and professionalism behaviours. However, more work must identify the best approaches to intervene early with underperforming learners in these domains and to mitigate the potential negative consequences of early labelling of a learner as ‘struggling’. (Guideline 25).

### Guideline 12.

*Do collect relevant data from multiple sources across case content ***(strong)**

Ideally, multisource feedback facilitates accurate identification and effective remediation more than a single-rater tool or informal workplace-based observation [117]. However, waiting to accumulate multiple pieces of evidence must be balanced against the risk of delayed identification of the struggling learner. Once a struggling learner is identified, usually as a result of an objective measure (for example, a failed exam) or a clinical teacher’s concern that a learner is not demonstrating the expected competency, it is imperative to review additional performance data. This review must be done with awareness of potential of implicit bias [118] and the fact that clinical competence is greatly impacted by case specificity and should therefore not be determined based on a single case [119]. Accordingly, when possible, we recommend multiple direct observations in more than one context (e.g. hospital, ambulatory clinic) across more than one clinical domain. It can also help if remediators have access to the academic records of the learner in order to assess for performance patterns.

### Guideline 13.

*Do explore multiple causes of learner struggle beyond educational or workplace issues ***(strong)**

It is common that non-academic factors contribute to or are a consequence of academic struggles. These include physical (new-onset medical conditions) and mental health issues (including psychiatric illness, personality disorders, substance abuse) and previously undiagnosed learning disabilities [78, 108]. Obviously, young adults are at risk of experiencing other stressors including juggling family and financial challenges, navigating cultural and community expectations, dealing with hierarchy, all the while learning to deal with the significant strains and constraints of medical training. In particular, junior medical learners must learn to manage their distress about and frustration with a chaotic and poorly organized healthcare delivery system and adjust to poorly perceived or understood learning environments [120], cognitive dissonance with ethical dilemmas [121, 122] and poor role modelling [94]. Assessment across multiple domains can also determine the overlap between skill deficits and attitudinal problems [123]. Because responses to stress can be adaptive or maladaptive [124, 125], only some learners facing these stressors may present with academic struggles.

International medical graduates, underrepresented minority trainees, older trainees, and trainees with prior failures are more likely to be identified as needing remediation [126]. Students from underrepresented minority groups in medicine are potentially at risk of stress from the consequences of discrimination. Underrepresented minority students in medicine report regular experiences of microaggressions as well as overt discrimination leading to unpleasant or harmful psychological impact [127]. Non-white candidates underperformed with respect to white candidates in the UK [128]. In the US, medical students who are older, have a child, or self-identify as Native American or Pacific Islander have more frequent ‘serious thoughts’ of dropping out. These groups are also at greater risk of academic problems with significant psychosocial stressors [129]. Similar patterns are seen in other contexts [130].

Independent of actual ability, underrepresented minority learners are at additional risk of underperforming in academic settings when they become anxious about confirming commonly held negative stereotypes about them. It has been our experience that this phenomenon, called stereotype threat [131], is operative in medical education. In a prior review of strategies for addressing struggling learners, Steinert speaks to this dynamic by explicitly asking, ‘whose problem is it?’ [18]. A range of systems-level and interpersonal interventions reduce the impact of stereotype threat, including raising awareness of this dynamic and restructuring assessments to avoid inadvertently reinforcing stereotypes. Until societal, institutional, and interpersonal interventions reduce discrimination, remediators must remain aware of these dynamics and design remediation strategies which address the critical underlying causes of underperformance [132], including advocacy for a student.

### Guideline 14.

*Do intervene proactively with struggling learners—do not rely on their initiative ***(strong)**

Even when the struggling learner is identified, early intervention may not follow [118]. Weaker learners inaccurately self-assess, tending to overrate themselves [99, 133]. In remediation programs specifically, only about 7% of struggling learners accurately self-referred to one guidance program; the majority of people in the program were high achievers with chronic anxiety about performance [3]. Academically weaker students and those suffering burnout tend to avoid seeking assistance [129, 134], so the system must do its best to identify and support these learners and the program must have the capacity, support, and willingness to compel struggling learners into remediation [6]. Students who accept remediation demonstrate longer-term improvement in test-taking than those who decline [135].

### Guideline 15.

*Do have trainees in remediation undergo intensive, longitudinal tutoring with emphasis on study skills, collaboratively designed plans, frequent high-quality feedback, and individualized assessment ***(strong)**

Most remediating students have multiple challenges and therefore generally do not respond to limited interventions, such as ‘teaching to the test’ [3, 7]. Successful interventions rely on a holistic approach that combines content development and improving self-regulated learning strategies. Specifically, such strategies target both cognitive and affective domains of learning, and focus on study skills using relevant academic content as exemplars [6, 7, 105, 136–138]. One common hallmark of these successful programs incorporates regular pre-arranged meetings to assess progress and achievement of goals, with high-quality feedback and assessments determining the need for mid-course corrections and/or consequences in the absence of acceptable improvement [6, 139, 140]. For medical knowledge remediation, ongoing regular facilitated small group work can enhance learners’ study skills using evidence-based strategies, such as retrieval rehearsal (Guideline 4), mixing content and types of problems in a given study session rather than focusing only on one subject or type of problem (‘interleaved practice’;[68]), generating explanations, and having students write their own multiple-choice questions [6, 7, 105, 137].

Data support the benefits of longitudinal intervention and follow-up. In non-medical settings, effective programs encompassed at least 12 sessions [139]. In pre-clerkship remediation interventions, Winston [6] found a strong enough dose effect to mandate attendance for a full semester in order to ensure success for up to 2 years: 15 or more sessions doubled the long-term pass rates over 10 or fewer sessions, a statistically significant finding consistent with other relevant studies [30, 104, 107, 137]. In addition to longer duration of remediation, longer-term follow-up leads to optimal outcomes [141, 142] (see also Guideline 18). Finally, increased faculty face time with struggling learners decreased the probability of probation (referral to administrative leadership due to unsuccessful remediation) by 3.1% per hour spent, and of all negative outcomes, by 2.6% per hour [3]. Of course, for pragmatic reasons, remediators must specify a time frame for expected improvement [94]. We emphasize that time is not the only component of worth—quality of the remediation interventions matters (see Guideline 26).

### Guideline 16.

*Do assess for and improve skills in learning self-regulation*** (strong)**

High-achieving students exhibit increased motivation [143] and have more awareness about how to effectively learn and cope with difficulty [144]. In contrast, the literature describes struggling students as typically not engaging in self-regulated learning, making inappropriate choices of learning strategies for written and clinical formats of assessment, and using maladaptive strategies for coping with failure. These maladaptive strategies include relying on rote memorization, adhering rigidly to prior strategies that previously worked in other contexts, emphasizing time and effort spent studying rather than actual knowledge acquisition and improvement of understanding, and externalizing reasons for failure [16, 99].

In struggling students, once the emotional reaction to failure passes, it is important to reframe failure as a normal, even expected or desirable outcome, in order to allow for readjustment of study approaches, re-examination of interaction challenges, and incorporation of improved techniques toward success. However, some learners externalize blame, which may manifest as an inability to process feedback, criticism of curriculum and assessment methods, dissatisfaction with programs that did not intervene earlier (and therefore are accountable for not upholding their implicit contract to teach effectively), and failure to seek formal support because it is viewed as policing. It is common for struggling learners to cycle through a range of these often contradictory negative attitudes as they come to terms with their predicament. Remediators who are able to establish a trusting relationship with the struggling learner can provide reality checks while providing empathic emotional guidance.

Remediation is most successful when remediators adopt a self-regulated learning perspective as a lens through which to view variations in learners’ beliefs and behaviours about remediation [145]. It is important to address a struggling learner’s self-efficacy with respect to remediating and pessimism about remediation, even if the learner expresses negative or openly defiant attitudes at first. Collaborative design of the remediation plan (for example, introducing evidence-based study strategies and encouraging students to select the course material to apply them to) supports learner autonomy [6]. Learners can develop their own formal remediation plans with personal reflections and specific strategies to gather evidence of improved performance [146]. We reiterate that because it takes many struggling learners time to accept their situation, as mentioned above, programs must not rely solely on learners’ motivations to initiate remediation. However, one study showed that even with mandatory remediation, participating learners can still report high self-motivation [7], suggesting that with patience, a supportive relationship, and time, these learners can strengthen their self-regulation skills and make progress.

Some residents and house officers may need extra support to develop the sophisticated level of self-regulation required to attain workplace efficiency and organization while also developing their clinical competence. DeKosky et al. [13] report in detail on a process and tools to help residents organize around common time-consuming tasks, including admitting a patient efficiently, performing effective pre-rounding, and composing daily progress notes and presentations.

Mild to moderate lapses in professionalism are common. In our experience, strategies that support self-regulation are the mainstay of the most effective remediation in these cases. Engaging learners in supportive, non-judgmental conversations about their behaviour with experienced individuals or a professionalism committee can impel deep and behaviour-altering self-reflection [147, 148]. Other well-publicized professionalism remediation practices include mandated mental health evaluation and critical reflection writing assignments [86]. Importantly, it is generally acknowledged that lapses in professionalism occur on a continuum and that markedly egregious unprofessional behaviour is much less likely to be remediable, especially if there is a pattern of unprofessional behaviour and evidence of serious characterological disorder. It is critical to consider each case individually [149].

### Guideline 17.

*Do remediate knowledge and skills in small groups with expert facilitators ***(moderate)**

Social cognitive theory posits that the effectiveness of group learning is based in discourse and development of critical thinking [150, 151]. Numerous examples of instructional designs in medical education are based on this theory, such as problem-based and team-based learning. Particularly in remediation, struggling students are often ‘unskilled and unaware’ [133] and may not recognize their own weaknesses. Seeing others with alternative solutions to similar challenges can help develop a sense of group identity and social regulation, which may in turn support self-regulation [152–154]. This group approach can help reduce stigma by emphasizing the pride of belonging, supporting each other, and feeling understood rather than isolated [102, 103]. Additional benefit arises when the group practises giving and receiving feedback. This develops lifelong skills of self-assessment, feedback, and possibly self-regulated learning. We note that while there is experience doing remediation for cognitive skills in small groups, there is no such evidence to support professionalism remediation using this strategy.

Expert faculty facilitation is crucial if remediation is to be done in groups. Skilful facilitation of small group learning, allowing for emotional support, explicit description and recognition of high quality work, and encouraging collaboration leads to success in both classroom-based as well as clinical skills remediation [6, 76, 103, 143, 153]. Trained faculty can prevent groupthink and premature closure of discussion, which is especially important for struggling students [93, 153]. In addition, faculty must highlight cognitive conflict and inconsistency, ask disruptive questions, and model intellectual curiosity [155, 156]. In general, learners prefer supervisors to be present to enhance their learning [156]; without guidance, they can develop bad habits and form ‘illusions of competence’ [157, 158].

### Guideline 18.

*Do follow-up with learners, even after the presumed end of the remediation period ***(moderate)**

The evidence is that for many (but not all) learners, underperformance is a pattern over time rather than an isolated easily resolvable problem [159]. This is likely multifactorial. An individual may initially have difficulty adjusting to the demands of medical education and training but ultimately acclimatize, or alternatively, never gain independent ability to accommodate to these demands. Only observation over time will tell. Additionally, even the most hardy learners have complex lives, and academic performance may fluctuate with non-academic demands on their time and energy. A supportive institutional culture encourages learners to self-monitor and seek help in adjusting to new challenges and invites private discussions about underachievement. These discussions can help both learners and faculty decide when and what type of support is necessary. For professionalism remediation, long-term engagement is needed to ensure that students have internalized new attitudes and skills.

## Don’ts

### Guideline 19.

*Don’t rely solely on quantitative academic markers of performance ***(moderate)**

The best predictor of academic performance in medical school is academic performance prior to medical school [160]; however, experiences and performance in medical school still matter. Though pre-admission aptitude tests and grade-point average account for approximately one-quarter of the variance in knowledge testing in medical school [128, 160–162], much room for growth and development remains within medical school. Given that there are scant long-term follow-up data showing that medical school applicants with higher grades become better physicians, providing remedial support on the basis of pre-application data alone is not justified, and may stigmatize some students. One study found that while performance on standardized academic metrics did not predict the need for remediation in the future, atypical characteristics of workplace assessments like ambiguous or negative comments or excessive length of text comments, did [163]. We believe emerging research will support these findings. Holistic programmatic portfolio-based assessment approaches hold some promise, although they are challenging to implement [164]. Multisource assessment data and competency or outcomes-based frameworks for medical education will likely provide much richer and more comprehensive data upon which to base academic coaching, promotion, and remediation judgments and a more reliable basis for the prediction of success in medical school and beyond.

### Guideline 20.

*Don’t merely give more time, repeat the learner experience, give general or vague advice, or just ‘teach to the test’ without additional support ***(strong)**

This guideline is essentially the opposite of guidelines 15–16. Merely decelerating a trainee without additional support does not significantly affect dismissal rates [165].

## Don’t knows

### Guideline 21.

*What are the long-term outcomes of remediation?*

We know very little about long-term outcomes of remediation programs. We do know that there was no longitudinal improvement in one 5‑year study, for students who initially failed OSCEs and then engaged in a standard remediation plan with short-term success [30]. Another case-control study of residents showed that remediating learners eventually reached competence levels similar to the mean but needed more exams and a longer time for completion [95]. Among students who failed a clinical performance exam, Klamen and Williams noted an improvement in post-remediation scores [96]. More of this longitudinal tracking of program outcomes is necessary to evaluate the efficacy of our interventions [166], and we strongly recommend long-term monitoring of students who have undergone remediation. This kind of prospective, longitudinal follow-up may highlight, for example, that remediation in medical school or residency predicts practice difficulties in the future. Additionally, given that trainees commonly move along the training path from one institutional context to another, tracking learner progress across such contexts may further illuminate the extent to which remediation practices and systems are institution-specific and longitudinally durable.

### Guideline 22.

*What is the optimal blend and duration of remediation?*

Especially for specific situations and difficulties, we do not know when to determine the completion of a learner’s remediation. Reasons for remediation vary for any given student, and there is rarely a single deficit. Given the complexity of remediation work, it is likely best conducted and assessed on a case-by-case basis.

That said, the educational evidence base supports that teaching struggling learners with a toolbox of approaches makes good sense. The key is to maximize the evidence-based approach for every component of a struggling learner’s remediation plan. These tend to be complex, highly individualized interventions. Therefore, it is difficult to tease out the impact of any specific element of the intervention, making general remediation rules elusive [138].

Though the weight of the data supports a dose effect (Guideline 15), it is unknown exactly how many interventions are necessary for optimal performance. Furthermore, a dose effect for clinical skills remediation is unclear, although the approach is quite similar to that of pre-clerkship remediation. In addition, it is possible that too many interventions may lead to a decrease in self-efficacy or independence. This is an area for future study.

### Guideline 23.

*How does remediation fit with CBME and its approach of learner-centredness and de-emphasis of time?*

Ideally, time-variable competency-based medical education (CBME) would make remediation as a separate educational activity irrelevant. However, trainees struggle for many reasons including, for example, ambivalence about career fit. Therefore, even in a fully realized CBME framework, there is likely to be a need for a ‘zone of remediation’ between the normal curriculum and exclusion [27]. This zone framework demonstrates how educational practice in different zones is based on different rules, roles and responsibilities. Thresholds for moving between zones would require explicit and transparent policies and specific expertise in remediation. While currently there are very few examples of truly time-variable CBME, it will be important to monitor challenges experienced by students in such a system to understand how policy and practise in the zone of remediation will need to evolve.

The move towards CBME brings with it the opportunity for an alternative paradigm to the current identify-and-intervene approach to remediation. However, to do so will require a shift in culture, from regarding those who take a little longer than others to achieve the required competencies as struggling to thinking about learning pace as an individual factor in ultimate success in practice. This is difficult to conceive of in systems which inherently remain time-based and competitive. CBME may provide an opportunity to consider ‘struggling’ learners with more positive regard and take a broader view of the many challenges that our learners encounter, while maintaining our obligation of high standards to society.

### Guideline 24.

*What is the optimal balance between the benefits of educational handovers and the need to protect learners from negative bias that may arise from such handovers? *(See Guideline 6 for further discussion.)

### Guideline 25.

*What specific measures predict the need for remediation?*

Several studies show that many quantifiable measures of performance carry neither reliable nor specific information to identify struggling learners early in medical training. Personality and study skills inventories add little to prediction of performance [114, 167] and are susceptible to faking [115]. Learning style assessments correlate weakly with academic performance [128], if at all [168, 169]. However, some indicators may be fruitful for future research. For example, one recent study found that systematic faculty ratings of in-class participation predicted failure of year 1 medical students before students began to underperform [170].

For clinical performance, USMLE Step 1 scores, part of the licensing exam taken early in medical school in the US, weakly predicted low clinical performance in medical school [171] and low knowledge; they did not predict professionalism issues in residency [172]. We believe that any further work to delineate some of these predictors must be balanced by the significant potential to stigmatize a student through early identification who would otherwise do well later in training. This paradox again emphasizes the utility of prospective, longitudinal studies.

### Guideline 26.

*Apart from establishing a longitudinal remediation program (Guideline 15), what are the most effective remediation practices?*

Few studies have explicitly attempted to delineate what components are necessary and sufficient for an effective remediation program. The wider remediation literature suggests that different things work for different people and that there is a complex relationship between individual and systems/organizational factors. Ultimately resources are limited, and the list of possible remediation strategies is long, highlighting the need for research that informs remediation policy and practices [17].

## Conclusion

Remediation is a highly complex process involving learners and faculty, individuals, systems and societal factors. The good news is that as medical educators, we have increasing awareness of and expertise in practices that can maximize educational outcomes for struggling learners. This paper summarizes what we currently know from the published literature and our own extensive experiences about remediation processes. We believe that whilst there is great need for ongoing improvement in this field, and whilst rigorous hypothesis testing in remediation studies remains challenging because of the ethical peril of a non-intervention condition, there is reason to be optimistic.

More than half of the ‘Do’s’ guidelines reach beyond individual interventions. These guidelines reflect the core values of education, highlighting the importance of expectation-setting and transparent educational policies and structures; balance of commitment to and compassion for our learners with our societal responsibility; the importance of a culture of feedback, due process, and non-judgmental positive regard for learners; proactivity when learners do not recognize their level of struggle; and a holistic approach to understanding the full range of causes when learners experience academic struggle. We aim to ensure that our guidelines underscore the importance of context, prevention, and early detection in this domain of medical education practice.

Most commonly, research in this domain has focused on testing the relationship between learners’ performance on a particular assessment and performances on later assessments and how a particular remediation program assists in helping a learner pass a specific examination. This type of work tends to obscure our understanding of the ways in which the context, learning environment, or an individual remediation intervention may lead to unintended consequences for certain individuals or groups. This limits our ability to make choices and understand trade-offs in remediation practice.

Remediation in medical education highlights the perennial tensions that pervade the field in general, and institutions and program leadership must navigate these tensions to ultimately make many complex and difficult decisions. What is the appropriate balance between providing resources to remediation and other important educational activities? How does a program determine when a learner in remediation is unlikely to succeed? What is the defensible balance between responsibility to society and ongoing support to a struggling learner who has already made high personal and financial sacrifice? What is any institution/program’s responsibility to address academic struggles whose causes lie wholly outside the purview of the institution (e.g., personal, family, illness)? Although research is desperately needed to guide this decision-making, educators working within the pragmatic limitations of institutions and programs must continue to answer these questions in the absence of clear data. Ultimately, decisions about remediation reflect institutional values, and therefore, clarifying those values is critical. Our hope is that this summary of the current state of remediation will enable individuals, institutions, and the medical profession to make more informed choices about how to support our struggling learners most effectively.
